# Complement lectin pathway protein levels reflect disease activity in juvenile idiopathic arthritis: a longitudinal study of the Nordic JIA cohort

**DOI:** 10.1186/s12969-019-0367-9

**Published:** 2019-09-09

**Authors:** Mia Glerup, Steffen Thiel, Veronika Rypdal, Ellen Dalen Arnstad, Maria Ekelund, Suvi Peltoniemi, Kristiina Aalto, Marite Rygg, Susan Nielsen, Anders Fasth, Lillemor Berntson, Ellen Nordal, Troels Herlin, for the Nordic Study Group of Pediatric Rheumatology (NoSPeR)

**Affiliations:** 1 Department of Pediatrics, Aarhus University Hospital, Palle Juul-Jensens Blvd. 99, 8200 Aarhus N, Denmark; 2 Department of Biomedicine, Aarhus University, Aarhus, Denmark; 3 Department of Pediatrics, University Hospital of North Norway, and Department of Clinical Medicine, UiT The Arctic University of Norway, Tromsø, Norway; 4 Department of Clinical and Molecular Medicine, NTNU - Norwegian University of Science and Technology, Trondheim, Norway; 5 Department of Pediatrics, Levanger Hospital, Nord-Trøndelag Hospital Trust, Levanger, Norway; 6 Department of Women’s and Children’s Health, Uppsala University, Uppsala, Sweden; 7 Department of Pediatrics, Ryhov County Hospital, Jonkoping, Sweden; 8 New Children’s Hospital, Pediatric Research Center, Helsinki University Central Hospital, University of Helsinki, Helsinki, Finland; 9 Department of Clinical and Molecular Medicine, NTNU - Norwegian University of Science and Technology, Trondheim, Norway; 10 Department of Pediatrics, St. Olavs Hospital, Trondheim, Norway; 11 Department of Pediatrics, Rigshospitalet, Copenhagen University Hospital, Copenhagen, Denmark; 12 Department of Pediatrics, Institute of Clinical Sciences, Sahlgrenska Academy, University of Gothenburg, Gothenburg, Sweden; 13 Department of Women’s and Children’s Health, Uppsala University, Uppsala, Sweden

**Keywords:** JIA, Lectin pathway proteins, Disease activity

## Abstract

**Background:**

To determine the serum levels of the lectin pathway proteins early in the disease course and 17 years after disease onset and to correlate the protein levels to markers of disease activity in participants from a population-based Nordic juvenile idiopathic arthritis (JIA) cohort. Additionally, to assess the predictive value of lectin pathway proteins with respect to remission status.

**Methods:**

A population-based cohort study of consecutive cases of JIA with a disease onset from 1997 to 2000 from defined geographical areas of Finland, Sweden, Norway and Denmark with 17 years of follow-up was performed. Clinical characteristics were registered and H-ficolin, M-ficolin, MASP-1, MASP-3, MBL and CL-K1 levels in serum were analyzed.

**Results:**

In total, 293 patients with JIA were included (mean age 23.7 ± 4.4 years; mean follow-up 17.2 ± 1.7 years). Concentrations of the lectin protein levels in serum were higher at baseline compared to the levels 17 years after disease onset (*p* ≤ 0.006, *n* = 164). At baseline, the highest level of M-ficolin was observed in systemic JIA. Further, high M-ficolin levels at baseline and at 17-year follow-up were correlated to high levels of ESR. In contrast, high MASP-1 and MASP-3 tended to correlate to low ESR. CL-K1 showed a negative correlation to JADAS71 at baseline.

None of the protein levels had prognostic abilities for remission status 17 years after disease onset.

**Conclusion:**

We hypothesize that increased serum M-ficolin levels are associated with higher disease activity in JIA and further, the results indicate that MASP-1, MASP-3 and CL-K1 are markers of inflammation.

## Background

Juvenile idiopathic arthritis (JIA), which is the most common rheumatic disease in childhood [1, 2], is a heterogeneous disease. The pathophysiology and etiology are multi-factorial and not fully understood. There is an increasing body of evidence that inadequately controlled activation of complement factors leading to either overactivity or deficiency may be involved in the pathogenesis of some autoimmune diseases [3–6]. However, the role of the complement system in JIA is still not fully elucidated [7–11]. Most studies involve investigations of the classical and the alternative pathway and have shown contradictory results [7, 9, 12–14]. The third initiating pathway of the complement system is the lectin pathway. This pathway is triggered by binding of one or more of the two collectin molecules mannose-binding lectin (MBL) and Collectin-LK (a heterodimer of the two polypeptide chains, Collectin-Liver 1 and Collectin-Kidney 1), or the ficolins (H-ficolin, L-ficolin and M-ficolin) to glycosylated surfaces on microbial cell walls or altered-self cells, normally confined to the immune system. Upon the binding to adequate patterns enzymatic proteins called MBL-associated serine proteases (MASPs) become activated [15–17]. Autoactivation of MASP-1 is followed by cleavage of MASP-2. The active protease of MASP-2 cleaves C4 and C2 forming a C4b2a convertase that subsequently cleaves C3. Consequently, activation of the common pathway eliminates target structures by initiation of membrane-attack complexes and inflammatory reactions. Active forms of MASP-3 activates Factor D which is a key enzyme of the alternative pathway in the complement system [18].

The relationship between the lectin dependent pathway and JIA is poorly understood.

Genetically determined deficiencies in some JIA categories have been investigated, and MBL polymorphisms are the most investigated variant alleles as they have been suggested to be associated with erosions and early onset of rheumatoid arthritis [19–21]. However, in JIA the results regarding MBL alleles have been contradictory. Gergely and colleagues [22] found that the MBL levels were lower in JIA than in controls and that there was an association between MBL gene mutations and predisposition to JIA, which is in contrast with the findings of Kang et al. [23].

Studies on the remaining lectin pathway proteins in JIA are scarce [11, 24]. In 2015, Petri et al. [24] compared the lectin pathway protein levels in patients with oligoarticular and systemic JIA (sJIA). They found that plasma levels of M-ficolin and MASP-2 were significantly higher in the sJIA group, which correlated positively to the levels of C-reactive protein (CRP) and erythrocyte sedimentation rate (ESR)). Additionally, M-ficolin levels correlated positively to the number of active joints. In JIA patients, Kasperkiewicz et al. [11] found no differences in the levels of M-ficolin or MASP-2 compared to controls but L-ficolin levels were lower in the oligoarticular JIA group. However, this was not related to clinical parameters.

Since the clinical implications of the lectin pathway proteins in JIA have previously been studied in very selected cohorts, we aimed to ponder the role of the lectin pathway in the non-selected, close to population-based Nordic JIA cohort. Specifically, our targets were to investigate the serum levels of lectin pathway proteins early in the disease course and 17 years after disease onset, to correlate with markers of disease activity and to explore the predictive capacity of the proteins with respect to achievement of remission.

## Materials and methods

### Study design

**Fig. 1:**
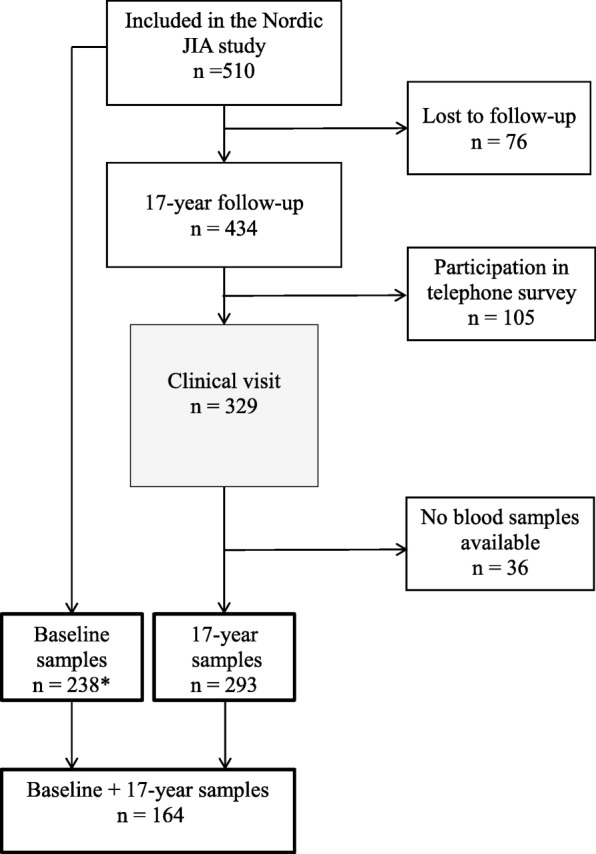
Flow chart of the study population. F = female, M = male, JIA = juvenile idiopathic arthritis. *The Finnish part of the cohort (*n* = 151) did not have any baseline samples taken

We performed a multicenter-based, prospective, observational study of participants from the close to population-based Nordic JIA cohort 17 years after disease onset. We included consecutive cases of newly diagnosed JIA patients from defined geographical areas of Denmark, Finland, Norway and Sweden, as previously described in detail [25]. Inclusion time was in the early biologic era from January 1st, 1997 to June 30th_,_ 2000. A baseline visit was aimed to take place 6 months (− 1/+ 2 months) after disease onset and prospectively follow-up thereafter [1, 26]. In the original cohort, 510 JIA patients were included; however, one center had no access to storage of blood samples at baseline and accordingly, this center was not included in comparison of baseline and 17-year data (Fig. 1). All eligible patients were invited to participate irrespectively of disease activity, level of treatment and disease course to ensure a non-selected setting. The 17-year follow-up visit included updating of the demographic data, a clinical examination and blood samples. We applied the juvenile arthritis disease activity score for 71 joints (JADAS71) [27] and the ACR 2011 criteria for disease inactivity and remission [28]*.*

### Inclusion criteria

Patients fulfilling the ILAR criteria [29] for JIA and having at least a baseline and a 17-year follow-up visit. There were no exclusion criteria.

### Protein assays

We measured H-ficolin, M-ficolin, MASP-1, MASP-3, MBL and collectin-K1 in serum at baseline and after 17 years. Because of limited serum volumes available, CL-L1, MASP-2 and MAp19 were not measured. L-ficolin was not measured as plasma samples are needed for test of this protein.

All samples were collected in serum tubes, centrifuged, aliquoted and stored at − 80 °C. Previously, lectins have been proven stable to repeated freeze/thaw cycles [30] and the baseline samples were thawed no more than two times. All concentrations were measured by validated in-house time-resolved immunofluorometric assay (TRIFMA) using a primary coat consisting of the relevant monoclonal antibody as previously described [30–35]. The principles of TRIFMA are similar to those of the enzyme-linked immunosorbent assay (ELISA) apart from the utilization of long-lasting fluorescence of europium as the labeling probe for the readout. To sum up, for the six proteins, diluted samples were incubated in microtiter wells coated with the relevant antibodies, binding the protein of interest to the antibody, and subsequently incubated with biotinylated antibodies and finally with europium-labeled streptavidin. The signal from the europium caught in the wells was read as time-resolved fluorometry. To ensure reproducibility three quality controls were added to each plate, and only when these values were below 15% variability the results of the plate were accepted.

### Ethics

This study was approved by the national research committees (1–10–72-280-13, 2012/2051, Dnr 2014/413–31, 174/13/03/03/2014), and all patients gave their written informed consent. Additionally, Institutional Review Board approval was granted.

### Statistical analysis

Descriptive statistics of mean and standard deviation (SD) or median and interquartile range (IQR) was used to assess the clinical characteristics of the cohort.

The concentrations of the six proteins were non-normally distributed, as evaluated by qq-plot and histogram. A χ^2^ test was used for comparison of dichotomous variables. The Mann–Whitney U test, Kruskal-Wallis test and Spearman’s rank correlation were used in the comparison of ordinal data. The Spearman’s rank correlation (rho) was defined as a strong correlation if ρ > 0.5, medium if 0.3 < ρ < 0.5 and weak if 0.1 < ρ < 0.3. Univariate and multivariate regression analyses were performed to assess the protein levels as baseline predictors of development of remission off medication or inactive disease 17 years after disease onset. The level of significance (p) was defined as ≤0.05.

## Results

### Study population

Of 510 eligible patients with a JIA onset from 1997 to 2000, blood samples were available in 238/510 at baseline. In 293/329 participants attending a clinical visit 17 years after disease onset blood samples were accessible. Among these 293 participants, additional blood samples from baseline were available in 164 participants (Fig. 1). The mean follow-up period was 17.2 ± 1.7 years (mean ± SD) after onset and the mean age of the study participants was 23.7 ± 4.4 years (mean ± SD) with 71% being females (Table 1). At baseline, ESR was significantly higher among participants with sJIA compared to the other JIA categories (median 27 mm/h and 14 mm/h, respectively; *p* = 0.03). Disease-modifying anti-rheumatic drugs (DMARDs) were prescribed in 45/238 (18.9%) cases at baseline. The distribution of JIA categories is described in Table 1. In general, the disease activity was low at the 17-year follow-up visit with a median active joint count of 0 (IQR 0–0) and 43% were clinically inactive with a JADAS71 ≤ 1. For further clinical characteristics at baseline and 17 years of follow-up, see Additional file 1: Table S1 and Additional file 2: Table S2. There was no significant difference in the age at onset or the distribution of JIA categories among the 293 participants with available blood samples at the 17-year follow-up compared to the 217 with no available blood samples. However, significantly more girls (*p* < 0.01) and a higher number of active joints at the baseline visit were found in the included group (*p* < 0.001).

**Table 1:** Clinical characteristics of participants in the Nordic JIA cohort at the 17-year follow-up visit

	Total cohort *N* = 293
Females, n (%)	208 (71.0)
Age at onset, y*	6.5 ± 4.1
Age at follow-up, y*	23.7 ± 4.4
Disease duration, y*	17.2 ± 1.7
ANA positive, n (%)	89 (30.4)
HLA-B27 positive, n (%)	66 (22.5)
CRP > 10 mg/L, n (%)	16 (5.4)
ESR > 20 mm/h, n (%)	18 (6.1)
Active joint count, median (IQR)	0 (0–0)
Cumulative joints, median (IQR)	8(4–15)
JADAS71 ≤ 1, n (%)	126 (43.0)
Systemic JIA	13 (4.4%)
Oligoarticular persistent	66 (22.5%)
Oligoarticular extended	55 (18.8%)
Polyarticular RF negative	53 (18.1%)
Polyarticular RF positive	5 (1.7%)
Psoriatic	19 (6.5%)
Enthesitis-related arthritis	33 (11.3%)
Undifferentiated	49 (16.7%)

y* mean in years ±SD, *ANA* antinuclear antibodies, *HLA-B27* human leucocyte antigen B27, *CRP* C-Reactive Protein, *ESR* Erythrocyte Sedimentation Rate, *IQR* 1st-3rd interquartile range, *JADAS71* juvenile arthritis disease activity score of 71 joints, *RF* rheumatoid factor

### Levels of the lectin pathway proteins at baseline and 17-year follow-up

We measured H-ficolin, M-ficolin, MASP-1, MASP-3, MBL and collectin-K1 levels in serum at baseline and at the 17-year follow-up and the results are shown in Table 2.

**Table 2:** Lectin protein concentrations according to JIA subtype early in disease course and at 17-year follow-up

	n	MBL	H-ficolin	M-ficolin	CL-K1	MASP-1	MASP-3
Baseline							
Total cohort	238	3.1(1.2–4.7)	26.3(21.5–29.9)	3.0(2.4–3.9)	0.28(0.23–0.34)	9.9(7.4–13.2)	8.1(6.7–9.3)
sJIA	11	3.8(0.8–4.7)	27.2(20.4–29.6)	4.1(3.4–6.3)	0.31(0.21–0.37)	7.4(4.8–12.1)	8.5(5.7–10.7)
Oligo persist	113	3.3(1.9–4.5)	25.8(21.8–29.9)	3.0(2.4–3.8)	0.28(0.23–0.34)	10.3(7.9–13.3)	8.1(7.2–9.6)
Oligo ext	8	3.1(1.2–4.4)	26.2(21.6–32.3)	3.5(2.3–4.8)	0.30(0.24–0.42)	7.0(5.7–11.5)	8.1(6.2–8.5)
Poly RF-	47	4.4(2.4–6.3)	26.7(21.2–35.3)	3.1(2.4–4.3)	0.28(0.23–0.33)	9.0(6.9–11.7)	7.7(6.4–9.0)
Poly RF+	5	2.3(2.3–4.6)	24.0(18.4–26.3)	2.8(2.4–2.8)	0.25(0.24–0.35)	8.8(8.5–10.7)	10.0(7.8–10.4)
Psoriatic	2	2.5(2.3–2.8)	24.5(20.4–28.6)	3.3(2.7–3.8)	0.23(0.19–0.26)	15.0(14.9–15.0)	8.2(5.6–10.9)
ERA	20	1.9(0.5–2.8)	26.4(21.9–29.4)	2.9(2.1–3.5)	0.26(0.20–0.33)	10.2(7.0–14.4)	8.1(6.5–9.0)
Undiff	32	2.2(0.5–4.7)	27.0(21.2–29.5)	3.0(2.6–3.5)	0.29(0.26–0.35)	10.6(8.5–3.6)	8.1(7.1–9.0)
17-year follow-up							
Total cohort	293	2.0(0.7–3.5)	22.5(18.9–26.1)	2.1(1.7–3.0)	0.24(0.21–0.28)	8.8(6.5–11.3)	6.7(5.6–8.4)
sJIA	13	3.3(1.6–3.8)	21.8(18.0–24.6)	2.2(1.6–2.4)	0.23(0.21–0.25)	8.2(7.7–10.5)	6.4(5.9–6.9)
Oligo persist	66	2.2(0.8–3.3)	22.1(18.8–25.6)	2.3(1.7–3.0)	0.24(0.21–0.27)	9.0(6.8–11.5)	6.6(5.1–8.6)
Oligo ext	55	2.0(0.6–3.3)	21.1(18.5–24.0)	2.0(1.7–2.7)	0.24(0.21–0.28)	9.5(7.5–13.0)	6.7(5.7–8.5)
Poly RF-	53	2.6(1.2–3.8)	22.3(18.8–25.4)	2.1(1.7–2.7)	0.24(0.22–0.28)	9.8(7.5–9.9)	6.7(5.7–8.4)
Poly RF+	5	1.3(0.0–2.2)	31.4(25.1–36.1)	2.1(1.8–3.3)	0.25(0.23–0.34)	8.4(6.3–10.9)	8.7(7.5–8.8)
Psoriatic	19	2.0(0.7–4.3)	24.9(21.8–30.3)	2.2(1.8–3.1)	0.27(0.22–0.28)	8.7(5.6–12.1)	6.6(5.7–9.1)
ERA	33	1.4(0.6–2.0)	25.4(22.1–30.6)	1.9(1.6–2.6)	0.24(0.20–0.27)	8.1(6.3–10.5)	7.2(5.7–8.9)
Undiff	49	1.9(0.6–3.6)	20.8(17.2–25.8)	2.4(1.9–3.3)	0.24(0.21–0.27)	8.6(6.4–11.7)	6.4(5.3–7.8)

Serum values are medians in μg/mL with 1st-3rd interquartile range (IQR). *MBL* mannan binding lectin, *MASP* MBL-associated serine proteases, *CL-K1* collectin kidney, *sJIA* systemic JIA, *Oligo persist* oligo persistent JIA, *Oligo ext.* oligo extended JIA, *Poly RF*- polyarticular rheumatoid factor negative JIA, *Poly RF+* polyarticular rheumatoid factor positive JIA, *ERA* enthesitis-related arthritis, *Undiff* undifferentiated JIA

**Fig. 2:**
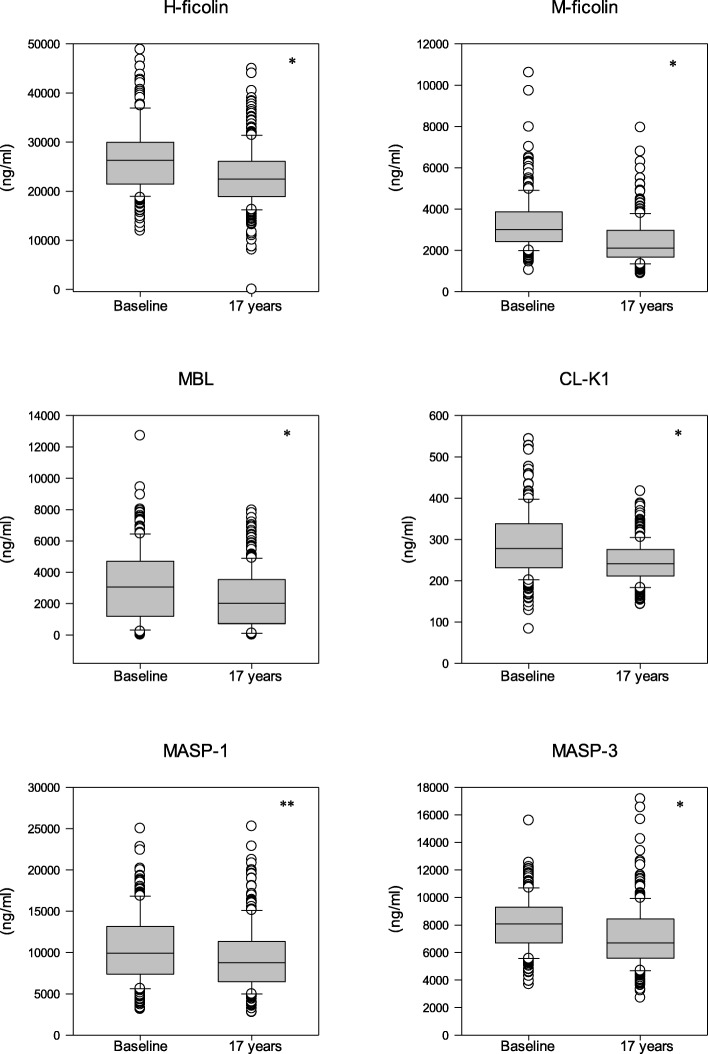
Differences in lectin pathway protein levels at baseline and 17 years of follow-up. MBL = mannan-binding lectin; MASP = MBL-associated serine proteases; CL-K1 = collectin kidney1. * *p* < 0.001; ** *p* = 0.001

Comparing the protein levels at baseline to the 17-year values showed significantly higher baseline levels for all proteins (Fig. 2, Wilcoxon, Z = -3.255 - -7.812, *p* = 0.006 or less). The reduction of serum levels was in the range of 12.5–30%, most pronounced for M-ficolin.

### Correlation between gender, JIA categories and lectin pathway levels

No gender difference in protein levels was found at baseline (*p* = 0.11–0.92).

At baseline highest levels of M-ficolin were found in the systemic group (median 4.1 μg/mL (IQR 3.4–6.3) (Table 2), which was significantly higher than in the oligoarticular persistent (*p* = 0.024), polyarticular RF-neg (*p* = 0.048), ERA (*p* = 0.02) and the undifferentiated category (*p* = 0.014). Conversely, MASP-1 levels at baseline were significantly lower for the systemic group (median 7.4 μg/mL (IQR (4.8–12.1)) compared to oligoarticular persistent (*p* = 0.03) and the undifferentiated category (*p* = 0.019). The other lectins did not differentiate significantly between the groups.

### Correlation between markers of disease activity, treatment, disease status and lectin pathway protein levels

The protein levels at baseline were compared to ESR, JADAS71 or cumulative joint count at baseline. M-ficolin was positively correlated to the ESR levels (Table 3); however, the correlation found was weak. At baseline, elevated ESR (> 20 mm/hr) was observed in 55/238 (23.1%) of patients investigated for lectin pathway proteins. Participants with elevated ESR had significantly higher M-ficolin levels than those with normal values (median 3.43 μg/mL (IQR 2.86–4.46) versus median 2.85 μg/mL (IQR 2.35–3.59) (*p* < 0.001)). Conversely, for MASP-1 and MASP-3 there was a tendency towards higher levels in patients with normal ESR compared to elevated ESR but the difference did not reach significance (*p* = 0.12 and *p* = 0.08, respectively) (data not shown).

**Table 3:** Correlation between disease activity and lectin levels at baseline and 17-years of follow-up

		BASELINE#(*n* = 238)		17-YEAR FOLLOW-UP (n = 293)		
	ESR	JADAS71	Cum joints	ESR	JADAS71	Cum joints
MBL	N.S.	N.S.	N.S.	N.S.	N.S.	N.S.
H-ficolin	N.S.	N.S.	N.S.	N.S.	N.S.	N.S.
M-ficolin	*r* = 0.23 p < 0.01	N.S.	N.S.	*r* = 0.20 p < 0.01	N.S.	N.S.
CL-K1	N.S.	*r* = −0.16 *p* = 0.05	N.S.	*r* = −0.13 *p* = 0.05	N.S.	N.S.
MASP-1	N.S.	N.S.	N.S.	N.S.	N.S.	N.S.
MASP3	N.S.	N.S.	*r* = −0.13 *p* = 0.04	N.S.	N.S.	N.S.

Only results with a *p* ≤ 0.05 are listed. *N.S* non-significant. #: Baseline was 6 months (−1/+ 2 months) after disease onset. *ESR* erythrocytes sedimentation rate at baseline, *JADAS71* juvenile arthritis disease activity score of 71 joints *cum joints* cumulative joint count, *MASP* MBL-associated serine proteases, *MBL* mannan-binding lectin, *CL-K1* collectin kidney, *r* Spearman’s rho

CL-K1 showed a weak negative correlation to JADAS71 at baseline (Table 3). In patients with inactive disease (JADAS71 ≤ 1) at 17 years of follow-up the serum M-ficolin levels were significantly lower than in patients with active disease (*p* = 0.026). In addition, MASP-3 correlated weakly to the cumulative joint count during the first 6 months after onset (Table 3).

Comparison of lectin levels 17 years after disease onset of patients in remission (on or off medication) according to the preliminary Wallace criteria versus not being in remission revealed no difference for any of the proteins (*p* = 0.10–0.78, *n* = 293) (data not shown).

None of the six proteins had significant explanatory power to predict remission off medication in the univariate regression analyses (Table 4). Likewise, the protein levels at baseline could not predict inactive disease 17 years after disease onset in a multivariate regression analysis (data not shown).

**Table 4:** Associations between lectin pathway proteins at baseline and remission 17 years after disease onset* (*n* = 238)

	Univariate crude odds ratio (95% CI)	*P* values
PREDICTORS EARLY IN DISEASE COURSE		
ESR	0.984(0.97–0.99)	0.01**
JADAS71	0.91(0.84–0.98)	< 0.01**
Cumulative joint count	0.92 (0.88–0.97)	< 0.01**
H-ficolin	0.86(0.38–2.25)	0.86
M-ficolin	1.53(0.69–3.42)	0.30
MASP-1	1.14(0.58–2.28)	0.70
MASP-3	0.87(0.26–2.93)	0.82
MBL	1.06(0.87–1.29)	0.56
CL-K1	1.69(0.62–4.60)	0.31

*according to the preliminary Wallace criteria, *ESR* erythrocytes sedimentation rate at baseline, *JADAS27* juvenile arthritis disease activity score of 27 joints; *cumulative joint count* cumulative joint count within the first 6 months (−1/+ 2 months) after disease onset, *MASP* MBL-associated serine proteases, *MBL* mannan-binding lectin, *CL-K1* collectin kidney 1, ** Statistical significance (*p* ≤ 0.05)

## Discussion

This is the first study to report lectin pathway protein levels related to JIA disease activity in a long-term follow up study of participants from the Nordic JIA cohort. The highest level of M-ficolin at baseline was observed in systemic JIA which was significantly higher than most of the other categories. Conversely, MASP-1 levels were significantly lower for the sJIA compared to the oligoarticular persistent group. We found that a high M-ficolin level at baseline and 17-year follow-up was correlated to high ESR and conversely, high MASP-1 and MASP-3 tended to correlate to low ESR. We found a tendency that high MASP-1 and MASP-3 were associated with low disease activity and CL-K1 was negatively correlated to JADAS71 at baseline. Accordingly, we hypothesize that the high levels of M-ficolin and low levels of MASP-1 and MASP-3 may reflect inflammation in JIA.

M-ficolin has previously been suggested to be involved in the pathogenesis of rheumatoid arthritis (RA) with the finding of a 30-fold increase in synovial fluid levels from patients with active RA compared to the concentrations in osteoarthritis [4]. M-ficolin is a protein found in the granules of monocytes and neutrophil granulocytes and may be released upon stimulation of the phagocytes [16]. Polymorphisms in the *FCN1* gene coding for M-ficolin have been described to be associated with the susceptibility to develop rheumatoid arthritis [36].

In DMARD-naïve patients with early rheumatoid arthritis (RA), increased circulating M-ficolin levels have been associated with higher disease activity, notably reflected by DAS28 and the HAQ, at both baseline and at 1 year [5]. Further, it was demonstrated that M-ficolin levels at baseline were the strongest predictor of remission and that baseline M-ficolin in the lowest quartile indicated a 95% chance of achieving low disease activity 1 year after diagnosis [5].

However, in the present study none of the baseline levels of lectin pathway proteins were able to predict disease outcome such as remission status 17 years after disease onset as otherwise suggested in previous studies in RA and JIA [5, 8].

An advancement of studies on the lectin pathway being a part of the pathogenesis in autoimmune diseases [6, 37], including JIA, could potentially lead to the identification of novel biomarkers. These biomarkers are of particular interest as they reflect more disease-specific information than the nonspecific acute-phase reactants available today (C-reactive protein (CRP) and erythrocyte sedimentation rate (ESR)). Understanding the molecular source for the JIA disease heterogeneity of JIA will be a milestone in identifying biomarkers of inflammation; markers that may prove valuable in therapeutic patient stratification and prediction of future disease behavior early in the disease course. Our findings suggest that increased circulating M-ficolin levels are associated with higher disease activity and presumably reflect biomarkers of inflammation in JIA. The lower levels of MASP-1 and MASP-3 when inflammatory activity is high may be suggestive of a consumption of activated enzymes, e.g. as a result of binding of the serpin C1-inhibitor to MASP-1 [38].

Consistent with our findings, Petri et al. [24] reported that M-ficolin levels were higher in patients with sJIA than oligo persistent JIA in a study of 128 children within the first year of disease course. Our data support the idea that innate immune mechanisms play an important role in sJIA [39, 40] and thus differs in its biology compared to the non-systemic JIA categories.

Kasperkiewicz et al. [11] found no difference between M-ficolin levels in the oligo- and polyarticular groups which also is coherent with the findings in our cohort.

A positive correlation between markers of disease activity and M-ficolin in JIA has also been demonstrated previously [24], and we can now add CL-K1 as another protein that correlates to disease activity. To our knowledge this is the first study to investigate this protein in JIA. Inversely, higher levels of MASP-1 and MASP-3 were found in the oligo persistent category compared to the sJIA category which is consistent with the findings of Petri et al. [24].

There have been several studies on the association of MBL deficiency and the susceptibility to JIA but the conclusions are contradictive [8, 11, 22, 41]. In 2017, Kasperkiewcz et al. [11] found no difference between MBL levels in the oligo- and polyarticular categories which is consistent with our results. The median MBL levels were lower compared to our findings, but no further interpretation can be made as they used a different method, and no clinical data on the disease duration, disease activity or treatment were provided. We found no correlation between MBL and disease activity markers suggesting that our findings cannot support the idea that MBL play a major role in the pathogenesis of JIA.

The population-based setting and the prospective design with paired follow-up samples closely related to measurements of disease activity are strengths of the present study.

A limitation of the study is the number of patients lost to follow-up and the small sample size in some of the JIA categories. Although being a small group the included 4.4% representing the sJIA category were comparable to the sJIA representation in other Nothern European, Western European and North American cohorts [42]. A concern could be the long-term stability of complement proteins over such a long storage period. Although we know storage for 5 years will have no influence on the measured levels (as indicated be the stability of the internal controls we keep in the freezer) the very long-term stability of the proteins is not fully elucidated and might have affected the results. Normal values for healthy children have not been studied previously; however, our baseline values are comparable to the levels found by Petri et al. [24]. We found no differences in age at onset or JIA categories between the participants and those lost to follow-up, but more girls and a higher number of active joints at baseline in the included group. The latter might have skewed the baseline levels of the proteins that are correlated to disease activity towards even higher levels. Further, the baseline samples were collected 6 months after disease onset, and almost 19% of the patients were on disease-modifying anti-rheumatic drugs when the samples were taken, which may have influenced the protein levels and conceivably have impaired the predictive abilities of the lectin protein as seen in RA studies [5].

## Conclusions

In summary, this study contributes with novel insights into the possible role of the lectin pathway in driving the ongoing inflammation in JIA although the exact mechanism is not completely understood. The results substantiate that M-ficolin is a marker of disease activity and additionally, MASP-1, MASP-3 and CL-K1 show weak correlations to changes in disease activity.; however, the levels of lectin pathway proteins measured early after disease onset could not anticipate the future disease course. Further studies in treatment naïve cohorts are needed to achieve a better understanding of the disease pathogenesis of JIA.

## Supplementary information

**Additional file 1: Table S1.** Clinical characteristics of participants in the Nordic JIA cohort at baseline. y* = mean in years ±SD,** = active joint count at the visit 6 months after onset (− 1/+ 2 months), *** = cumulative joint count during the first 6 months (− 1/+ 2 months) after disease onset, ****DMARDs = Disease modifying anti-rheumatic drugs at the baseline visit, ANA = antinuclear antibodies, HLA-B27 = human leucocyte antigen B27, CRP = C-Reactive Protein, ESR = Erythrocyte Sedimentation Rate, IQR = 1st-3rd interquartile range, JADAS71 = juvenile arthritis disease activity score of 71 joints, sJIA = systemic JIA, Oligo persist = oligo persistent JIA, Oligo ext. = oligo extended JIA, Poly RF- = polyarticular rheumatoid factor negative JIA, Poly RF+ = polyarticular rheumatoid factor positive JIA, ERA = enthesitis-related arthritis, Undiff = undifferentiated JIA. (DOCX 17 kb)

**Additional file 2: Table S2.** Clinical characteristics of participants in the Nordic JIA cohort at the 17-year follow-up visit. y* = mean in years ± SD, ANA = antinuclear antibodies, HLA-B27 = human leucocyte antigen B27, CRP = C-Reactive Protein, ESR = Erythrocyte Sedimentation Rate, IQR = 1st-3rd interquartile range, JADAS71 = juvenile arthritis disease activity score of 71 joints, sJIA = systemic JIA, Oligo persist = oligo persistent JIA, Oligo ext. = oligo extended JIA, Poly RF- = polyarticular rheumatoid factor negative JIA, Poly RF+ = polyarticular rheumatoid factor positive JIA, ERA = enthesitis-related arthritis, Undiff = undifferentiated JIA. (DOCX 17 kb)

## Data Availability

The datasets generated and/or analyzed during the current study are not publicly available for ethical reasons, as well as privacy reasons, but are available from the Nordic Study group of Pediatric Rheumatology (NoSPeR) on reasonable request.

## Abbreviations

CL-K1: Collektin Kidney 1
CRP: C-reactive protein
DMARDs: disease-modifying anti-rheumatic drugs
ELISA: enzyme-linked immunosorbent assay
ESR: Erythrocyte sedimentation rate
IQR: interquartile range
JADAS71: juvenile arthritis disease activity score for 71 joints
JIA: Juvenile Idiopathic Arthritis
MASP: MBL-associated serine proteases
MBL: mannose-binding lectin
RA: Rheumatoid arthritis
rho: Spearman’s rank correlation
SD: Standard deviation
sJIA: systemic JIA
TRIFMA: time-resolved immunofluorometric assay

## References

[1] Berntson L, Andersson Gare B, Fasth A, Herlin T, Kristinsson J, Lahdenne P (2003). Incidence of juvenile idiopathic arthritis in the Nordic countries. A population based study with special reference to the validity of the ILAR and EULAR criteria. J Rheumatol.

[2] Harrold LR, Salman C, Shoor S, Curtis JR, Asgari MM, Gelfand JM (2013). Incidence and prevalence of juvenile idiopathic arthritis among children in a managed care population, 1996-2009. J Rheumatol.

[3] Medjeral-Thomas NR, Troldborg A, Constantinou N, Lomax-Browne HJ, Hansen AG, Willicombe M (2018). Progressive IgA nephropathy is associated with low circulating Mannan-binding lectin-associated serine Protease-3 (MASP-3) and increased glomerular factor H-related Protein-5 (FHR5) deposition. Kidney Int Rep.

[4] Ammitzboll CG, Thiel S, Ellingsen T, Deleuran B, Jorgensen A, Jensenius JC (2012). Levels of lectin pathway proteins in plasma and synovial fluid of rheumatoid arthritis and osteoarthritis. Rheumatol Int.

[5] Ammitzboll CG, Thiel S, Jensenius JC, Ellingsen T, Horslev-Petersen K, Hetland ML (2013). M-ficolin levels reflect disease activity and predict remission in early rheumatoid arthritis. Arthritis Rheum.

[6] Troldborg A, Thiel S, Trendelenburg M, Friebus-Kardash J, Nehring J, Steffensen R (2018). The lectin pathway of complement activation in patients with systemic lupus erythematosus. J Rheumatol.

[7] Gilliam BE, Reed MR, Chauhan AK, Dehlendorf AB, Moore TL (2011). Significance of complement components C1q and C4 bound to circulating immune complexes in juvenile idiopathic arthritis: support for classical complement pathway activation. Clin Exp Rheumatol.

[8] Dolman KM, Brouwer N, Frakking FN, Flato B, Tak PP, Kuijpers TW (2008). Mannose-binding lectin deficiency is associated with early onset of polyarticular juvenile rheumatoid arthritis: a cohort study. Arthritis Res Ther..

[9] Brunner J, Prelog M, Riedl M, Giner T, Hofer J, Wurzner R (2012). Analysis of the classical, alternative, and mannose binding lectin pathway of the complement system in the pathogenesis of oligoarticular juvenile idiopathic arthritis. Rheumatol Int.

[10] Prokopec KE, Berntson L, Oman A, Kleinau S (2012). Up regulated complement and fc receptors in juvenile idiopathic arthritis and correlation with disease phenotype. J Clin Immunol.

[11] Kasperkiewicz K, Eppa L, Swierzko AS, Bartlomiejczyk MA, Zuber ZM, Siniewicz-Luzenczyk K (2017). Lectin pathway factors in patients suffering from juvenile idiopathic arthritis. Immunol Cell Biol.

[12] Miller JJ, Olds LC, Silverman ED, Milgrom H, Curd JG (1986). Different patterns of C3 and C4 activation in the varied types of juvenile arthritis. Pediatr Res.

[13] Jarvis JN, Pousak T, Krenz M, Iobidze M, Taylor H (1993). Complement activation and immune complexes in juvenile rheumatoid arthritis. J Rheumatol.

[14] Aggarwal A, Bhardwaj A, Alam S, Misra R (2000). Evidence for activation of the alternate complement pathway in patients with juvenile rheumatoid arthritis. Rheumatology (Oxford).

[15] Worthley DL, Bardy PG, Mullighan CG (2005). Mannose-binding lectin: biology and clinical implications. Intern Med J.

[16] Endo Y, Matsushita M, Fujita T (2011). The role of ficolins in the lectin pathway of innate immunity. Int J Biochem Cell Biol.

[17] Degn SE, Thiel S (2013). Humoral pattern recognition and the complement system. Scand J Immunol.

[18] Dobo J, Kocsis A, Gal P (2018). Be on target: strategies of targeting alternative and lectin pathway components in complement-mediated diseases. Front Immunol.

[19] Jacobsen S, Madsen HO, Klarlund M, Jensen T, Skjodt H, Jensen KE (2001). The influence of mannose binding lectin polymorphisms on disease outcome in early polyarthritis. TIRA Group J Rheumatol.

[20] Graudal NA, Homann C, Madsen HO, Svejgaard A, Jurik AG, Graudal HK (1998). Mannan binding lectin in rheumatoid arthritis. A longitudinal study. J Rheumatol.

[21] Graudal NA, Madsen HO, Tarp U, Svejgaard A, Jurik G, Graudal HK (2000). The association of variant mannose-binding lectin genotypes with radiographic outcome in rheumatoid arthritis. Arthritis Rheum.

[22] Gergely P, Pazar B, Nagy ZB, Gombos T, Rajczy K, Balogh Z (2009). Structural polymorphisms in the mannose-binding lectin gene are associated with juvenile idiopathic arthritis. J Rheumatol.

[23] Kang H, Chen T, Li H, Xu Q, Cao S, Wei S (2017). Prognostic factors and disease course in aquaporin-4 antibody-positive Chinese patients with acute optic neuritis. J Neurol.

[24] Petri C, Thiel S, Jensenius JC, Herlin T (2015). Investigation of complement-activating pattern recognition molecules and associated enzymes as possible inflammatory markers in Oligoarticular and systemic juvenile idiopathic arthritis. J Rheumatol.

[25] Glerup M, Rypdal V, Arnstad ED, Ekelund M, Peltoniemi S, Aalto K, et al. Long-term outcomes in juvenile idiopathic arthritis: 18 years of follow-up in the population-based Nordic juvenile idiopathic arthritis (JIA) cohort. Arthritis Care Res (Hoboken). 2019. 10.1002/acr.23853 30762291

[26] Nordal E, Zak M, Aalto K, Berntson L, Fasth A, Herlin T (2011). Ongoing disease activity and changing categories in a long-term nordic cohort study of juvenile idiopathic arthritis. Arthritis Rheum.

[27] Consolaro A, Ruperto N, Bazso A, Pistorio A, Magni-Manzoni S, Filocamo G (2009). Development and validation of a composite disease activity score for juvenile idiopathic arthritis. Arthritis Rheum.

[28] Wallace CA, Ruperto N, Giannini E, Childhood A, Rheumatology Research A (2004). Pediatric Rheumatology international trials O, et al. preliminary criteria for clinical remission for select categories of juvenile idiopathic arthritis. J Rheumatol.

[29] Petty RE, Southwood TR, Manners P, Baum J, Glass DN, Goldenberg J (2004). International league of associations for Rheumatology classification of juvenile idiopathic arthritis: second revision, Edmonton, 2001. J Rheumatol.

[30] Troldborg A, Hansen A, Hansen SW, Jensenius JC, Stengaard-Pedersen K, Thiel S (2017). Lectin complement pathway proteins in healthy individuals. Clin Exp Immunol.

[31] Krarup A, Sorensen UB, Matsushita M, Jensenius JC, Thiel S (2005). Effect of capsulation of opportunistic pathogenic bacteria on binding of the pattern recognition molecules mannan-binding lectin, L-ficolin, and H-ficolin. Infect Immun.

[32] Wittenborn T, Thiel S, Jensen L, Nielsen HJ, Jensenius JC (2010). Characteristics and biological variations of M-ficolin, a pattern recognition molecule, in plasma. J Innate Immun.

[33] Degn SE, Jensen L, Gal P, Dobo J, Holmvad SH, Jensenius JC (2010). Biological variations of MASP-3 and MAp44, two splice products of the MASP1 gene involved in regulation of the complement system. J Immunol Methods.

[34] Thiel S, Moller-Kristensen M, Jensen L, Jensenius JC (2002). Assays for the functional activity of the mannan-binding lectin pathway of complement activation. Immunobiology..

[35] Selman L, Henriksen ML, Brandt J, Palarasah Y, Waters A, Beales PL (2012). An enzyme-linked immunosorbent assay (ELISA) for quantification of human collectin 11 (CL-11, CL-K1). J Immunol Methods.

[36] Vander Cruyssen B, Nuytinck L, Boullart L, Elewaut D, Waegeman W, Van Thielen M (2007). Polymorphisms in the ficolin 1 gene (FCN1) are associated with susceptibility to the development of rheumatoid arthritis. Rheumatology (Oxford).

[37] Das N (2015). Complement and membrane-bound complement regulatory proteins as biomarkers and therapeutic targets for autoimmune inflammatory disorders, RA and SLE. Indian J Exp Biol.

[38] Parej K, Dobo J, Zavodszky P, Gal P (2013). The control of the complement lectin pathway activation revisited: both C1-inhibitor and antithrombin are likely physiological inhibitors, while alpha2-macroglobulin is not. Mol Immunol.

[39] Pardeo M, Bracaglia C, De Benedetti F (2017). Systemic juvenile idiopathic arthritis: new insights into pathogenesis and cytokine directed therapies. Best Pract Res Clin Rheumatol.

[40] Nigrovic PA (2014). Review: is there a window of opportunity for treatment of systemic juvenile idiopathic arthritis?. Arthritis Rheumatol.

[41] Kang M, Wang HW, Cheng PX, Yin ZD, Li XO, Shi H (2006). Lack of association between mannose-binding lectin gene polymorphisms and juvenile idiopathic arthritis in a Han population from the Hubei province of China. Arthritis Res Ther.

[42] Consolaro A, Giancane G, Alongi A, van Dijkhuizen EHP, Aggarwal A, Al-Mayouf SM (2019). Phenotypic variability and disparities in treatment and outcomes of childhood arthritis throughout the world: an observational cohort study. Lancet Child Adolesc Health.

