# Supplementary material for: Complement lectin pathway protein levels reflect disease activity in juvenile idiopathic arthritis: a longitudinal study of the Nordic JIA cohort
Source: Pediatr Rheumatol Online J. 2019 Sep 9;17:63. doi: 10.1186/s12969-019-0367-9 (PMC6734250; doi:10.1186/s12969-019-0367-9)
Supplement: Supplementary file 1 — Additional file 1: Table S1. Clinical characteristics of participants in the Nordic JIA cohort at baseline. y* = mean in years ±SD,** = active joint count at the visit 6 months after onset (− 1/+ 2 months), *** = cumulative joint count during the first 6 months (− 1/+ 2 months) after disease onset, ****DMARDs = Disease modifying anti-rheumatic drugs at the baseline visit, ANA = antinuclear antibodies, HLA-B27 = human leucocyte antigen B27, CRP = C-Reactive Protein, ESR = Erythrocyte Sedimentation Rate, IQR = 1st-3rd interquartile range, JADAS71 = juvenile arthritis disease activity score of 71 joints, sJIA = systemic JIA, Oligo persist = oligo persistent JIA, Oligo ext. = oligo extended JIA, Poly RF- = polyarticular rheumatoid factor negative JIA, Poly RF+ = polyarticular rheumatoid factor positive JIA, ERA = enthesitis-related arthritis, Undiff = undifferentiated JIA. (DOCX 17 kb) [file 12969_2019_367_MOESM1_ESM.docx]

**Additional file 1: Table S1** Clinical characteristics of participants in the Nordic JIA cohort at baseline.

| n (%) | Total cohort  238 | sJIA  11 (4.6) | Oligo  persistent  113 (47.5) | Oligo  extended  8 (3.4) | Poly RF-  47 (19.7) | Poly RF+  5 (2.1) | Psoriatic  2 (0.8) | ERA  20 (8.4) | Undiff  32 (13.4) |
| --- | --- | --- | --- | --- | --- | --- | --- | --- | --- |
| Females, n (%) | 166 (69.7) | 7 (63.6) | 78 (69.0) | 8 (100) | 35 (74.5) | 5 (100) | 1 (50.0) | 5 (25.0) | 27 (84.4) |
| Age at onset, y* | 6.8±4.3 | 6.0±5.4 | 6.1±3.9 | 6.1±5.0 | 5.8±3.9 | 13.4 ±0.9 | 4.4 ±1.7 | 10.1±3.1 | 8.3±4.4 |
| Age at visit, y* | 7.5±4.3 | 6.6±5.4 | 6.8±3.9 | 7.3±4.7 | 6.4±3.9 | 13.9±0.9 | 4.9±1.7 | 10.9±3.1 | 9.1±4.3 |
| ANA positive, n (%) | 54 (22.7) | 0 (0.0) | 32 (28.3) | 3 (37.5) | 14 (29.8) | 0 (0.0) | 0 (0.0) | 1 (5.0) | 4 (12.5) |
| HLA-B27 positive, n (%) | 44 (18.5) | 0 (0.0) | 14 (12.4) | 2 (25.0) | 8 (17.0) | 0 (0.0) | 0 (0.0) | 14 (70.0) | 6 (18.8) |
| CRP >10 mg/L,n (%) | 49 (32.8) | 8 (72.7) | 16 (25.7) | 3 (37.5) | 14 (48.9) | 0 (20.0) | 0 (50.0) | 5 (30.0) | 3 (21.9) |
| ESR >20 mm/h,n (%) | 55 (23.1) | 7 (63.6) | 19 (16.8) | 5 (62.5) | 14 (29.8) | 0 (0.0) | 0 (0.0) | 6 (30.0) | 4 (12.5) |
| Active joint count,  median (IQR)** | 1 (0–18) | 1 (0–2) | 1 (0–4) | 3 (1–4) | 3 (0–5) | 4 (2–8) | 2 (1–3) | 2 (0.5–3.5) | 2 (1–5) |
| JADAS71≤1, n (%)  Cumulative joints,  median (IQR)*** | 24 (10.1)  3 (1-7) | 1 (9.1)  4 (2-7) | 16 (14.2)  2 (1-3) | 0 (0.0)  7.5 (5-11.5) | 3 (6.4)  8 (6-15) | 0 (0.0)  11 (10-14) | 0 (0.0)  4 (2-6) | 1 (5.0)  5 (2-8.5) | 3 (9.4)  3.5 (1-10) |
| DMARDs **** | 45 (18.9) | 5 (45.5) | 1 (0.9) | 4 (50.0) | 21 (44.7) | 2 (40.0) | 1 (50.0) | 6 (30.0) | 5 (15.6) |

y*= mean in years ±SD,**= active joint count at the visit 6 months after onset (-1/+2 months), ***= cumulative joint count during the first 6 months (-1/+2 months) after disease onset, ****DMARDs= Disease modifying anti-rheumatic drugs at the baseline visit, ANA= antinuclear antibodies, HLA-B27= human leucocyte antigen B27, CRP= C-Reactive Protein, ESR= Erythrocyte Sedimentation Rate, IQR= 1^st^-3^rd^ interquartile range, JADAS71= juvenile arthritis disease activity score of 71 joints, sJIA = systemic JIA, Oligo persist =oligo persistent JIA, Oligo ext= oligo extended JIA, Poly RF- =polyarticular rheumatoid factor negative JIA, Poly RF+ = polyarticular rheumatoid factor positive JIA, ERA= enthesitis-related arthritis, Undiff = undifferentiated JIA.
