# Supplementary material for: Complement lectin pathway protein levels reflect disease activity in juvenile idiopathic arthritis: a longitudinal study of the Nordic JIA cohort
Source: Pediatr Rheumatol Online J. 2019 Sep 9;17:63. doi: 10.1186/s12969-019-0367-9 (PMC6734250; doi:10.1186/s12969-019-0367-9)
Supplement: Supplementary file 2 — Additional file 2: Table S2. Clinical characteristics of participants in the Nordic JIA cohort at the 17-year follow-up visit. y* = mean in years ± SD, ANA = antinuclear antibodies, HLA-B27 = human leucocyte antigen B27, CRP = C-Reactive Protein, ESR = Erythrocyte Sedimentation Rate, IQR = 1st-3rd interquartile range, JADAS71 = juvenile arthritis disease activity score of 71 joints, sJIA = systemic JIA, Oligo persist = oligo persistent JIA, Oligo ext. = oligo extended JIA, Poly RF- = polyarticular rheumatoid factor negative JIA, Poly RF+ = polyarticular rheumatoid factor positive JIA, ERA = enthesitis-related arthritis, Undiff = undifferentiated JIA. (DOCX 17 kb) [file 12969_2019_367_MOESM2_ESM.docx]

**Additional file 2: Table S2** Clinical characteristics of participants in the Nordic JIA cohort at the 17-year follow-up visit.

| n (%) | Total cohort  293 | sJIA  13(4.4) | Oligo  persistent  66(22.5) | Oligo  extended  55(18.8) | Poly RF-  53(18.1) | Poly RF+  5(1.7) | Psoriatic  19(6.5) | ERA  33(11.3) | Undiff  49(16.7) |
| --- | --- | --- | --- | --- | --- | --- | --- | --- | --- |
| Females, n (%) | 208 (71.0) | 9 (69.2) | 48 (72.7) | 45 (81.8) | 39 (73.6) | 4 (80.0) | 15 (78.9) | 10 (30.3) | 38 (77.6) |
| Age at onset, y* | 6.5±4.1 | 5.9±4.0 | 6.0±3.6 | 4.8±3.8 | 5.8±4.1 | 10.9±2.3 | 6.4±4.1 | 9.5±3.4 | 7.6±4.1 |
| Age at follow-up, y* | 23.7±4.4 | 23.9±4.2 | 23.3±3.8 | 22.0±4.0 | 22.8±4.4 | 28.7±2.2 | 23.6±4.5 | 26.7±4.0 | 24.9±4.5 |
| Disease duration, y* | 17.2±1.7 | 18.0±0.7 | 17.3±1.1 | 17.3±1.4 | 17.0±1.3 | 17.8±1.0 | 17.2±0.9 | 17.1±2.7 | 17.3±2.6 |
| ANA positive, n (%) | 89 (30.4) | 3 (23.1) | 18 (27.2) | 22 (40.0) | 15 (28.3) | 2 (40.0) | 5 (26.3) | 8 (24.2) | 16 (32.7) |
| HLA-B27 positive, n (%) | 66 (22.5) | 2 (15.4) | 6 (9.1) | 8 (14.5) | 7 (13.2) | 1 (20.0) | 3 (15.8) | 26 (78.8) | 12 (24.5) |
| CRP >10 mg/L,n (%) | 16 (5.4) | 0 (0.0) | 2 (3.0) | 3 (5.5) | 3 (5.7) | 0 (0.0) | 2 (10.5) | 4 (12.1) | 2 (4.1) |
| ESR >20 mm/h, n (%) | 18 (6.1) | 0 (0.0) | 3 (4.5) | 3 (5.5) | 3 (5.7) | 0 (0.0) | 3 (15.8) | 3(9.1) | 3 (6.1) |
| Active joint count,  median (IQR) | 0 (0–0) | 0 (0–0) | 0 (0–0) | 0 (0–1) | 0 (0–1) | 0 (0–1) | 0 (0–0) | 0 (0–1) | 0 (0–0) |
| JADAS71≤1, n (%) | 126 (43.0) | 9 (69.2) | 37 (56.1) | 20 (36.4) | 29 (54.7) | 2 (40.0) | 6 (31.6) | 8 (24.2) | 15 (30.6) |
| Cumulative joints,  median (IQR) | 8(4-15) | 7(4-12) | 2(1-3) | 9(7-12) | 14(8-24) | 17(15-20) | 9(5-13) | 12(7-20) | 10(4-22) |

y*= mean in years ± SD, ANA= antinuclear antibodies, HLA-B27= human leucocyte antigen B27, CRP= C-Reactive Protein, ESR= Erythrocyte Sedimentation Rate, IQR= 1^st^-3^rd^ interquartile range, JADAS71= juvenile arthritis disease activity score of 71 joints, sJIA= systemic JIA, Oligo persist= oligo persistent JIA, Oligo ext= oligo extended JIA, Poly RF- = polyarticular rheumatoid factor negative JIA, Poly RF+ = polyarticular rheumatoid factor positive JIA, ERA= enthesitis-related arthritis, Undiff = undifferentiated JIA.
